# Comparison of the outcomes between thoracoscopic and laparoscopic esophagectomy via retrosternal and prevertebral lifting paths by the same surgeon

**DOI:** 10.1186/s12957-017-1219-z

**Published:** 2017-08-30

**Authors:** Bing Lv, Yong-Zhong Tao, Yu Zhu, Jing Wu, Bin Zhong, Fu-Chao Luo, Yang Liu, Ze-Xue Zhang

**Affiliations:** Department of Cardiothoracic Surgery, Fuling Central Hospital, NO 2, Gaosuntang Road, Fuling District, Chongqing, Fuling 408000 China

**Keywords:** Esophageal cancer, Chest laparoscopic, Esophagectomy, Retrosternal path, Path, Prevertebral

## Abstract

**Background:**

The objective of the study is to explore the effects of retrosternal and prevertebral lifting paths of the tubular stomach on postoperative complications of patients undergoing cervical anastomosis in thoracoscopic and laparoscopic esophagectomy.

**Methods:**

Sixty-three patients were retrospectively analyzed. The patients received thoracoscopic and laparoscopic esophagectomy by the same surgeon. According to the path by which the stomach was lifted upward, the patients were divided into two groups: the retrosternal path group (32 patients) and the prevertebral path group (31 patients). Operative indications and complications of postoperative patients in these two groups were observed.

**Results:**

There was no statistically significant difference in the time duration of surgery, amount of bleeding, number of dissected lymph node, and postoperative hospitalization time between the retrosternal and prevertebral lifting paths (*P* > 0.05). Furthermore, the two groups did not show significant difference in the incidence rate of postoperative anastomosis fistula complications (*P =* 0.702*)*. Instead, the amount of postoperative gastric drainage and the incidence rates of the pulmonary infection were significantly lower in the retrosternal path group than in the prevertebral path group, respectively (*P* = 0.001, *P* = 0.012, respectively).

**Conclusion:**

The esophagogastrostomic cervical anastomoses performed via the retrosternal and prevertebral paths are both feasible methods of digestive tract reconstruction. The amount of postoperative gastric drainage volume and the pulmonary infection incidence rate in the retrosternal path group were lower than those in the prevertebral path group. Therefore, gastroesophageal anastomosis via the retrosternal lifting path may be preferably considered for thoracoscopic and laparoscopic surgery for esophageal carcinoma patients.

## Background

Esophageal cancer is the sixth most common cause of cancer-related death worldwide [1]. The highest risk area for this cancer is often referred to as the “esophageal cancer belt,” stretching from northern Iran through the central Asian republics to North-Central China, and 90% of cases are squamous cell carcinomas [2, 3]. Minimally invasive esophagectomy (MIE) has the potential to reduce the morbidity and mortality of esophageal cancer surgery. Open surgery is the current standard treatment; however, it is a complex procedure with high morbidity and mortality. To overcome this problem, MIE was introduced in the early 1990s and has yielded good postoperative results (morbidity 4%; mortality 1%) [4]. In addition, a previous study reported that the tubular stomach reconstruction approach prevents postoperative recurrence and metastasis of esophageal cancer. Therefore, we adopted the thoracoscopic and laparoscopic cervical esophagogastric anastomosis technique in esophageal carcinoma patients to reconstruct the tubular stomach [5].

However, most of the conclusions in literatures are based on data collected by different surgeons, which increased influences induced by subjective factors. Therefore, in order to reduce the impact of surgeon-subjective factors on these results, it may be valuable that these data be collected from the same surgeon in order to analyze the influences of retrosternal and prevertebral lifting paths of the tubular stomach on operative complications of patients with esophageal cancer. In this study, the clinical data of 63 patients who underwent thoracoscopic and laparoscopic esophagectomy and anastomosis of the esophagus and stomach were retrospectively analyzed. All operations were conducted by the same surgeon in the method of gastric tube reconstruction in the Thoracic Surgery Department of our hospital from January 2013 to December 2015, and our experience and outcomes were presented.

## Methods

### General data

Inclusion criteria: From January 2013 to October 2015, a total of 63 patients underwent surgery operated by the same surgeon in xxx hospital. All patients were pre-operatively diagnosed with middle and upper esophageal squamous cell carcinoma by gastroscopy. In addition, upper gastrointestinal barium meal, as well as preoperative chest and abdominal enhanced computed tomography (CT), was performed to exclude distant metastases. Furthermore, electrocardiogram (EKG), pulmonary function, and blood biochemical examinations were performed to evaluate the tolerance of patients of the operation. All the patients with esophageal carcinoma underwent esophagus tumor resection and left cervical esophagogastric anastomosis on the right chest and abdomen via thoracoscopy and laparoscopy. According to different paths, in which the stomach was lifted in cervical gastric anastomosis, the patients were divided into two groups: the retrosternal path group (32 patients) and the prevertebral path group (31 patients). The difference in gender, age, body weight, tumor location, clinical stage, and postoperative pathological stage between these two groups were not statistically significant (*P* > 0.05), but were comparable (Table 1).

**Table 1:** Comparison of clinical data of the two groups

Clinical data	Esophageal bed pathway group (*n* = 31)	Substernal pathway group (*n* = 32)	*p* (value)
Sex (m/f)	25/7	23/8	0.757
Age (years)	61.6 ± 7.4	60.4 ± 7.2	0.684
Locations of esophageal			
Upper	8(25.8%)	10(31.2%)	0.632
Middle	23(74.2%)	22(68.8%)	0.596
Periods in TNM before surgery			0.975
T_1_N_0_M_0_	2 (6.5%)	3 (9.4%)	
T_1_N_1_M_0_	1 (3.2%)	1 (3.2%)	
T_2_N_0_M_0_	8 (26.8%)	7 (21.8%)	
T_2_N_1_M_0_1	3 (9.7%)	2 (6.3%)	
T_3_N_0_M_0_	13 (41.9%)	14 (43.7%)	
T_3_N_1_M_0_	4 (12.9%)	5 (15.6%)	

### Surgical methods

All patients underwent double lumen endotracheal tube intubation, through the application of intravenous combined anesthesia; esophagus tumor resection and cervical esophagogastric anastomosis were implemented via the three incisions of thoracoscopy through the right chest, abdomen, and left neck. For these two groups, in addition to the different paths where the stomach was lifted to the neck, the azygos vein arch was retained in the retrosternal path, while it was cut off routinely in the prevertebral path; the rest of the surgical procedures were basically the same.

#### Thoracoscopic surgery

The patients assumed a 90° left lateral decubitus position. The surgeon positioned on the patient’s right, the observation hole was selected at the seventh intercostal space in the anterior axillary line, two 0.5-cm main operation holes were located between the fourth and seventh intercostal spaces in the posterior axillary line, and the secondary operation hole was selected in the fourth intercostal space of the anterior axillary line with a length of approximately 2 cm. These were mainly used for the placement of the lung retractor, to press the lungs into the ventral side and expose the mediastinum. A clear operation field was maintained through suction, the esophagus was exposed and pulled out, and the thoracic cavity was explored to find the metastasis. The mediastinal pleura was opened along the esophagus using an electric coagulation hook combined with an ultrasonic scalpel to explore any obvious invasion to the esophagus. The esophagus located on both sides of the azygos vein was separated first. For the prevertebral path group, two ends of the azygos vein were clipped off with a biological clip and cut off with an ultrasonic scalpel. For the retrosternal path group, the azygos vein was clipped off, but not cut off. The esophagus was separated downward to the esophageal hiatus and disarticulated at the esophageal hiatus with an Endo-GIA. Then, the esophageal stump was clamped with an oval clamp to stretch upward, the esophagus was separated using an ultrasonic scalpel upward along the esophageal posterior wall to the inlet of the thorax, the bilateral laryngeal recurrent nerve was protected, the esophagus was disarticulated at the thoracic entrance, the esophageal carcinoma was excised, and the lymph nodes were dissected aside at the inferior pulmonary vein, carina of trachea, esophagus bed, and superior mediastinum, especially at the bilateral laryngeal recurrent nerve chain. In order to completely stop the bleeding, the chest was washed, the bronchial membrane and other places were checked to find any leakage, the chest drainage tube was placed into the observation hole, the incisions were closed, and double lung ventilation was restored.

#### Laparoscopic surgery

Patients assumed the herringbone position, with the head at a high position and the foot at a low position, and a small pillow was padded under the shoulders. First, a 1-cm incision was made under the left costal margin. Then, the skin and subcutaneous tissue were cut open, a laparoscope was placed after pneumoperitoneum, and two 1-cm main operation holes were located in the bilateral paraumbilical areas. The surgeon operated the ultrasonic scalpel with the right hand, while the left hand operated the separating forceps. There were two secondary operation holes. One hole was located at 0.5 cm under the subxiphoid, and the other hole was located at 0.5 cm to the partial lateral of the right lower costal margin. These were used by the assistant to stretch the stomach. The surgeon operated at the perineal side of the patient, the assistant stood at the right side of the patient, and the one who held the laparoscope stood at the left side of the patient. Abdominal cavity exploration was done to determine whether there was adhesion in the cavity and whether there were nodules and lumps in the liver, spleen, pelvic cavity, and the great omentum. Omental tissue of the greater gastric curvature was separated along the lateral gastroepiploic vessel arch by using an ultrasonic scalpel. Then, the gastrocolic ligament, left gastroepiploic artery, short gastric artery, and pericardial blood vessels were cut off. Separation of the lesser curvature of the stomach was done by transecting the ligament of the liver and stomach, protecting the right gastric vessel, upward lifting the stomach, pulling the separated left gastric vessel at the pancreatic upper margin, clipping off the vessel with a biological clip and cutting off with a ultrasonic scalpel, and dissecting the lymph nodes of the left gastric artery, splenic artery, and the common hepatic artery. The thoracic peritoneum located at the esophageal and diaphragmatic hiatus was separated and opened to free the stomach. For the prevertebral path group, the two sides of the crural diaphragm were partly mutilated. This operation was not required for the retrosternal path group. The pneumoperitoneum was canceled, the subxiphoid port was extended with an approximately 3–4-cm incision, the stomach was stretched out of the abdominal cavity, a tubular stomach with a diameter of approximately 3–4 cm was produced with a straight-type cutting stapler, and the cutting margin of the interrupted seromuscular layer was closed. A mark was made by sewing with a 7-0 silk thread on top of the stomach bottom, and the stomach was reversed into the abdominal cavity with no torsion infallibly.

#### Neck surgery

A 4-cm incision was made at the leading edge of the sternocleidomastoid muscle of the left neck, the cervical esophagus was separated along the inner side of the cervical vaginae vasorum, the lymph adipose tissue beside the laryngeal recurrent nerve in the tracheoesophageal groove was dissected and connected to the chest, and the esophagus was held up. The stapler nail mat was implanted after the cervical esophagus was transected. For the retrosternal path, the thoracic outlet was fully expanded after separating the retrosternal tunnel, and the tubular stomach was sent to the left neck. For the prevertebral path, the tubular stomach was pulled from the esophagus to the left neck by a traction wire. The digestive tract was reconstructed by end-to-side anastomosis with the stapler. The Endo-GIA cutting closure device was used to treat the gastric stump and the tube was placed near the pylorus, complete hemostasis was achieved, and the incision was closed after placement of a 2-ml negative pressure drainage tube at the neck incision.

#### Jejunostomy

Jejunostomy was performed for the two groups of patients, the gastrointestinal decompression tube was placed at postoperation routinely, and the postoperative treatment principle was the same.

### Statistical analysis

Measurement and count data were collected in this study. SPSS 17.0 statistical software was adopted for statistical analysis, and measurement data were presented as mean ± standard deviation ($\overline{x}\kern0.5em \pm \kern0.5em \mathrm{SD}$). Count data were presented as a proportion (percentage), independent sample *t* test was used to compare the measurement data, and the count data were analyzed by chi-square test. *P* < 0.05 was considered statistically significant.

## Results

All of the patients underwent esophagectomy, and there were no mortality and no cases which were converted into open chest surgery during surgery.

### Operation index

Operation time, blood loss during operation, number of dissected lymph nodes, and time of hospitalization were compared between the two groups; the difference was not statistically significant (*P* > 0.05). However, gastric drainage volume after surgery was significantly lower in the retrosternal path group than in the prevertebral path group (210 ± 120 ml vs. 730 ± 150 ml, *P* = 0.001). Surgical indicators in the two groups are shown in Table 2.

**Table 2:** Analysis of the two groups’ clinical results after surgery ($\overline{x}\kern0.5em \pm \kern0.5em \mathrm{s}$)

Index	Esophageal bed pathway group (*n* = 31)	Substernal pathway group (*n* = 32)	*p* (value)
Operation time (min)	244 ± 42	260 ± 50	0.482
Intraoperative blood loss (ml)	155 ± 55	150 ± 60	0.553
Quantities of lymph node cleared out (per case)	15 ± 9	14 ± 10	0.142
Quantities of gastric juice drainage for 5 days before surgery (ml)	730 ± 150	210 ± 120	0.001
Duration in hospital (days) after surgery	13 ± 4	13 ± 7	0.128

Note: all patients did not receive adjuvant chemoradiotherapy before surgery

### Analysis of surgical complications

Incidence rates of postoperative anastomotic leakage, arrhythmia, gastric emptying disorder, and other complications were compared between the two groups; the differences were not statistically significant (*P* > 0.05). However, the incidence rate of pulmonary infection was significantly lower in the retrosternal path group than in the prevertebral path group (*P* = 0.012). The operation index of the two groups is shown in Table 3.

**Table 3:** The condition of complications during the perioperative period in the two groups [cases (%)]

Complications	Esophageal bed pathway group (*n* = 31)	Substernal pathway group (*n* = 32)	*p* (value)
Anastomotic fistula	2 (6.5%)	2 (6.3%)	0.702
Pulmonary infection	7 (22.6%)	2 (6.3%)	0.012
Functional delayed gastric emptying	2 (6.5%)	1 (3.2%)	0.059
Arrhythmia	1 (3.2%)	1 (3.2%)	0.108
Others	3 (9.7%)	2 (6.3%)	0.285

## Discussion

Esophageal carcinoma is a common malignant tumor of the digestive tract in China, and surgical resection is known to be curative for locoregional disease in the case of esophageal cancer [6]. Three-incision subtotal esophagectomy was recommended for esophageal cancer surgery especially for squamous cell carcinoma of the thoracic esophagus [7]. Nevertheless, the conventional open esophagectomy has the disadvantages of extensive trauma, intense pain, and slow recovery. Video-assisted thoracoscopic surgery (VATS) shows an overall benefit on short-term quality of life (QOL) for the patients with esophageal cancer during the follow-up of 6 months after esophagectomy, compared with open surgery [8]. The basis of minimally invasive techniques in esophageal surgery is to maintain the therapy effectiveness and quality of traditional operations, while reducing perioperative injury. It also can provide superior visualization and magnified view than open esophagectomy to perform thoracoabdominal esophagectomy with three-field lymphadenectomy to prevent lymph node metastasis [9]. With the continuous improvement of minimally invasive endoscopic techniques, thoracoscopic and laparoscopic esophagectomies are not only suitable for patients with early esophageal cancer but are also suitable for parts of T3 patients [10]. The study conducted by Zingg et al. [11] has also shown that minimally invasive esophagectomy could reduce the incidence of pulmonary complications and respiratory failure in patients undergoing esophageal carcinoma surgery. Currently, the postoperative outcomes of retrosternal or prevertebral lifting paths of tubular stomach were controversial [12, 13]. In our study, the different paths of stomach lifting in these two kinds of cervical esophagogastric anastomosis were completed by the same surgeon. This research method may be more valuable for the evaluation of the prognosis and reduce human disturbances in aspects of operation style, method of anesthesia, position, and whether artificial pneumothorax and other aspects as far as possible during surgery.

The surgical effect of the retrosternal and prevertebral paths in the recession of esophageal carcinoma is one of the subjects in a clinical study [14]. Both the retrosternal and prevertebral paths are commonly used paths for tubular stomach upward lifting at present. Some scholars believe that the use of the prevertebral path has advantages of short anastomosis distance and operating convenience [15]. Furthermore, as this operation can be completed without separating the gap, intraoperative bleeding risk is small and incidence rates of postoperative anastomosis fistula, anastomotic stenosis, and other complications are low. However, other scholars believe that the isolation of the retrosternal gap is safe, fast, and very convenient for stomach lifting. In addition, the retrosternal path in conventional three-incision surgery can be quickly and easily operated, operation time is not significantly different from the prevertebral path, the separation of the retrosternal gap does not significantly increase the amount of bleeding, and the postoperative radiotherapy does not influence the reconstruction of the digestive tract [16–18]. In this study, operation time, bleeding volume, the number of dissected lymph nodes, and length of hospital stay between the two groups did not show significant differences. These findings revealed that the procedure of separating the gap was not a determinant factor for operation time and bleeding losses.

It was also observed that the volume of gastric juice drainage was significantly less in the retrosternal path group than in the prevertebral path group, and this result is consistent with a previous study [19]. Previous studies have indicated that the incidence rates of duodenal reflux in patients who were operated through the retrosternal path were significantly lower than patients who were operated through the prevertebral path [20, 21]. The reason for this may be because the sternum and pericardium provide continuous pressure on the tubular stomach of patients in the retrosternal path group, reducing gastric and duodenal reflux. We also believed that there were certain angles between the tubular stomach and thoracic outlet, as well as in the abdominal cavity, in patients from Shantou Hospital affiliated to Shantou University in the retrosternal path group, which could reduce gastric reflux and drainage [22]. In addition, respiratory movement and the heart beat may promote peristalsis of the tubular stomach to a certain extent, which is beneficial to the gastric emptying. Therefore, postoperative reflux symptoms were lighter in patients operated through the retrosternal path than in patients operated through the prevertebral path; this presented with lower postoperative gastric drainage volume compared to patients in the prevertebral path group. Furthermore, this might be related to the preservation of the azygos vein arch during operation through the retrosternal path.

Anastomotic leakage, anastomotic stenosis, pulmonary infection, respiratory failure, arrhythmia, and thoracic infection are common complications after resection of esophageal carcinoma [23]. Anastomotic leakage and anastomotic tension are closely associated with blood transport function. Some scholars found that the incidence rate of postoperative anastomotic leakage was higher in patients who were operated through the retrosternal path than in patients who were operated through the prevertebral path [24]. It was reported that the retrosternal path was slightly longer than the prevertebral path. Furthermore, the tension of the anastomosis was larger, and both oxygen and blood supply to the anastomotic stoma were more insufficient compared to the prevertebral path. Moreover, the incidence rate of anastomotic leakage was higher. However, in this study, postoperative anastomotic leakage rate was low in these two groups and the difference was not significant. We speculate that this may be related to the following factors: (1) both groups with a tubular stomach diameter of 4 cm resulted in the effective increasing length of the residual stomach, and no obvious tension at the anastomotic stoma was found during the operation; (2) attention should be given in reducing the compression of the sternum outlet in the stomach. We expanded the width of the upper thoracic opening to 6–8 cm, and the gastric remnant was in a hypotonic state that made the blood vessels more smooth and reduced the incidence of anastomotic fistula; (3) a 2-mm negative pressure suction tube was placed at the cervical incision after operation, which kept the surrounding of the anastomosis clean and promoted the healing of the anastomotic stoma; and (4) the thin gastric tube was used before the operation, and indwelling time of the gastrointestinal decompression tube was extended, which kept the stomach in the empty state and reduced the impact of the anterior mediastinum in the stomach and anastomotic stoma, thereby effectively reducing the occurrence of fistula. In this study, the time of gastrointestinal decompression in most patients was more than 6 days, and the negative pressure drainage at the neck incision was helpful in reducing the incidence of anastomotic leakage. In addition, preoperative parenteral nutrition was given to patients in both groups to maintain normal levels of plasma protein; postoperative enteral nutrition support treatment was strengthened via jejunostomy fistulas to promote postoperative anastomotic stoma healing. Those factors might contribute to the absence of significant differences in anastomotic leakage between the two groups.

Pulmonary and thoracic infections are common complications after resection of esophageal cancer, and these are also the main causes of the reduced effect of surgery and length of stay. In recent years, studies have shown that thoracoscopic esophageal cancer resection can shorten recovery time, reduce lung function damage, reduce the incidence rate of postoperative pulmonary complications, and achieve the same effect as open chest surgery [25]. The study conducted by Taguchi et al. [26] revealed that thoracoscopic and laparoscopic esophageal cancer resection could better protect lung function and improve the quality of life of patients. In this study, postoperative pulmonary complications were significantly less in patients in the retrosternal path group than in patients in the prevertebral group, which is consistent with previous studies [23]. The reason for it might be when the routine retrosternal tunnel was separated, the surgeon expanded the superior thoracic aperture and the tubular stomach was sent to the neck position on the left when the routine retrosternal tunnel was separated in the retrosternal path group, providing maximum maintenance in thoracic cavity volume and lung function [20]. In addition, thoracoscopic incision was small with light pain and the integrity of the thorax was retained; therefore, there was little impact on the respiratory function of patients. In addition, this is conducive to early postoperative sputum drainage, thereby reducing the incidence of pulmonary infection. Therefore, the incidence rates of postoperative pulmonary infection were low in patients who underwent these two kinds of paths.

In summary, cervical esophagogastric anastomoses performed through different stomach lifting paths (retrosternal and prevertebral paths) are both feasible for digestive tract reconstruction. Postoperative gastric drainage volume and the incidence rate of pulmonary infection were less in patients in the retrosternal path group compared to patients in the prevertebral path group. Gastroesophageal anastomosis via the retrosternal path can be considered as a priority. However, further studies on surgical complications, long-term follow-ups, and survival rate are needed.

## Conclusion

The esophagogastrostomic cervical anastomoses performed via the retrosternal and prevertebral paths are both feasible methods of digestive tract reconstruction. Postoperative gastric drainage volume and pulmonary infection incidence rate in the retrosternal path were lower than those in the prevertebral path. Gastroesophageal anastomosis via the retrosternal path may be preferably considered for thoracoscopic and laparoscopic surgery for esophageal carcinoma patients.

## Abbreviations

MIE: Minimally invasive esophagectomy
